# A genetic analysis of dragonfly population structure

**DOI:** 10.1002/ece3.4255

**Published:** 2018-06-25

**Authors:** Payton Phillips, Bradley J. Swanson

**Affiliations:** ^1^ Department of Biology Central Michigan University Mt. Pleasant Michigan

**Keywords:** amplified fragment‐length polymorphisms, dispersal, *Sympetrum obtrusum*, temporal cycling

## Abstract

Dragonflies reside in both aquatic and terrestrial environments, depending on their life stage, necessitating the conservation of drastically different habitats; however, little is understood about how nymph and adult dragonflies function as metapopulations within connected habitat. We used genetic techniques to examine nymphs and adults within a single metapopulation both spatially and temporally to better understand metapopulation structure and the processes that might influence said structure. We sampled 97 nymphs and 149 adult *Sympetrum obtrusum* from eight locations, four aquatic, and four terrestrial, at the Pierce Cedar Creek Institute in Southwest Michigan over two summers. We performed AFLP genetic analysis and used the Bayesian analysis program STRUCTURE to detect genetic clusters from sampled individuals. STRUCTURE detected *k* = u4 populations, in which nymphs and adults from the same locations collected in different years did not necessarily fall into the same clusters. We also evaluated grouping using the statistical clustering analyses NMDS and MRPP. The results of these confirmed findings from STRUCTURE and emphasized differences between adults collected in 2012 and all other generations. These results suggest that both dispersal and a temporal cycle of emergence of nymphs from unique clusters every other year could be influential in structuring dragonfly populations, although our methods were not able to fully distinguish the influences of either force. This study provides a better understanding of local dragonfly metapopulation structure and provides a starting point for future studies to investigate the spatial and temporal mechanisms controlling metapopulation structure. The results of the study should prove informative for managers working to preserve genetic diversity in connected dragonfly metapopulations, especially in the face of increasing anthropogenic landscape changes.

## 1 INTRODUCTION

The disparate requirements between their nymph and adult stages mean that dragonflies are dependent on both terrestrial and aquatic habitats, which have been increasingly modified in the face of anthropogenic activity. Despite increasing alteration and destruction of both terrestrial and aquatic habitats, relatively little is understood about the genetic relationship between populations of nymph and adult dragonflies in a given area. Previous studies have generally examined either the spatial distribution of dragonfly nymphs (e.g., Benke & Benke, 1975; Buskirk, 1987; Pierce, Crowely, & Johnson, 1985) or the movement of adults (e.g., Conrad, Willson, Harvey, Thomas, & Sherratt, 1999; Dolný, Harabiš, & Mižičová, 2014; Remsburg, Olson, & Samways, 2008). A better understanding of the connections between nymph and adult populations is essential to understanding dragonfly conservation and population structure.

Within natal ponds, factors including predation, competition, and nymph population density can impact the survival and growth of nymphs, thereby limiting the emergence of adult dragonflies (Benke & Benke, 1975; Buskirk, 1987; Pierce et al., 1985). After emerging from natal ponds, sexually immature dragonflies will move away from the water to forage, later returning to an aquatic habitat to reproduce (Conrad et al., 1999; Remsburg et al., 2008). The movement away from their natal location to forage may also provide immature dragonflies with an opportunity to disperse to different breeding sites prior to reproduction. In areas where nymph and adult population densities are low, adults from surrounding populations may move into the area to utilize available resources and find mates (Dempster, Atkinson, & French, 1995). Thus, it is possible that there may be little genetic relationship between the adults breeding at a site and the nymphs at the same site.

Due to the organization of dragonfly habitat into distinct bodies of water within or near sometimes patchily distributed terrestrial foraging habitat, dragonfly populations likely exist as either isolated populations or metapopulations (Suhonen et al., 2010). In the case of metapopulations, structure may be influenced by migration of immature individuals for foraging, dispersal of adults for breeding purposes, and semivoltinism of dragonfly nymphs within natal ponds. Studies which have examined population structure of dragonflies using nymph and adult specimens have not looked directly at these mechanisms (Monroe & Britten, 2014). Dispersal within the subpopulations of a metapopulation affects population size, persistence, spatial distribution, gene flow, and adaptation to the local environment (Kleinhans & Jonsson, 2011). Dispersal can be key to regional survival, as each subpopulation within a metapopulation faces local extirpation if dispersal is halted (Elkin & Possingham, 2008; Heinz, Wissel, & Frank, 2006). Subsequent recolonization can restore the subpopulations, but results in a reduction in genetic diversity, eventually increasing the likelihood of the metapopulation going extinct (Austin, Ovaskainen, & Hanski, 2011). Additionally, dispersal is often biased toward high‐quality habitats, leading to increased extinction in low‐quality habitats, especially when dispersal rates are low (Nee & May, 1992).

For *Sympetrum* species, previous studies have suggested that adult dispersal rates, specifically moving away from the natal water to breed, can vary from <10% to almost 100%, depending on the species (Dolný, Mizicova, & Harabis, 2013). In smaller areas with higher connectivity between subpopulations, it is unclear if dispersal between ponds remains low and adults return to the natal source of water to breed, or if they breed elsewhere. Methods which only evaluate short‐term movement of dragonflies may confound the migration of immature dragonflies for foraging with adult breeding dispersal; however, analyses of metapopulation structure through genetic techniques can elucidate long‐term relationships within a metapopulation (Keller, Brodbeck, Flöss, Vonwil, & Holderegger, 2010).

In addition to adult movement, metapopulation structure may be influenced by temporal factors such as the ability of nymphs to overwinter for multiple years. Dragonflies of many species are semivoltine, meaning that they take more than a single year to complete their life cycle. Voltinism is more common at higher latitudes and is influenced by the permanency of available natal habitat, as well as by photoperiod and temperature (Corbet, 1999; Corbet, Suhling, & Soendgerath, 2006). The semivoltine life history results in overlapping generations to some degree in populations of adult dragonflies (Kormondy & Gower, 1965). Alternatively, semivoltinism also may result in different generations emerging each year and rotating between years such that similar genetic populations emerge from natal ponds every other year (Pintor & Soluk, 2006). Thus, genetic population structure of dragonflies may be the result of both spatial movement patterns and temporal patterns of adult emergence.

In this study, we examine the metapopulation structure of adult and nymph dragonflies of the species *Sympetrum obtrusum* over 2 years. We analyze the genetic structure and differentiation of individuals from several small ponds and terrestrial foraging areas within a 2 km^2^ area to better understand dragonfly metapopulation structures at a local level. Because of the variety of processes potentially influencing metapopulation structure, we examine the population through both spatial and temporal lenses.

## 2 MATERIALS AND METHODS

### 2.1 Sampling

**Figure 1:**
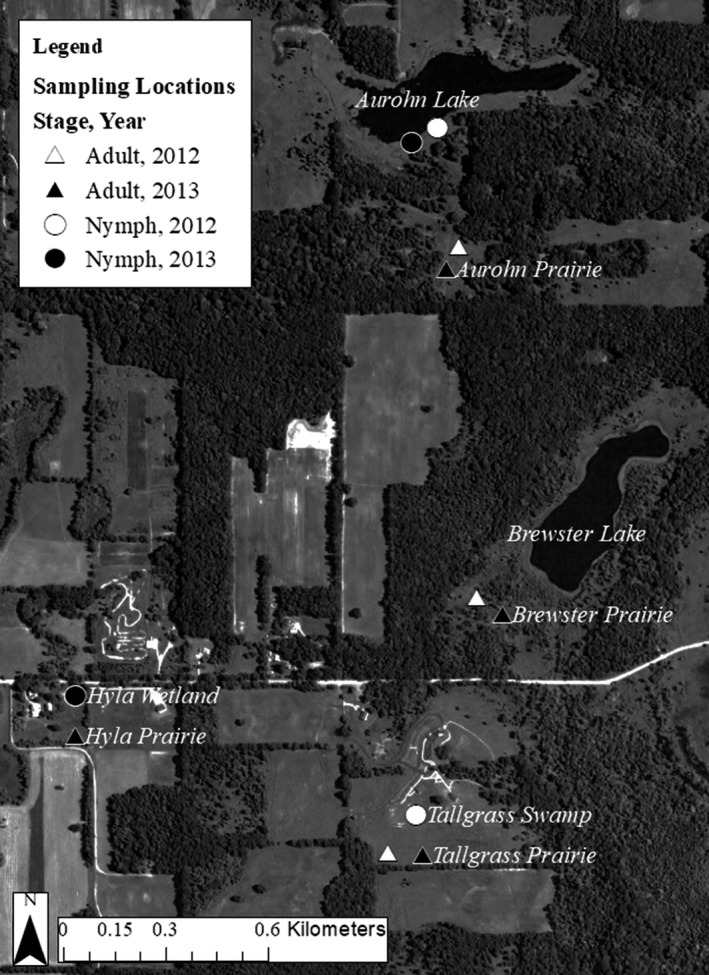
Map of the study area, indicating sampling locations by year and targeted life stage of *Sympetrum obtrusum*. Nymphs were sampled in wetlands and bodies of water, while adults were collected in adjacent prairies. Sampling locations for adults are indicated by triangles, while sampling locations for nymphs are indicated with circles. Samples collected in 2002 are coded in white, and samples from 2013 are coded in black. The satellite basemap from ESRI shows land cover (a mosaic of primarily forest and farmland), roads, and water

We collected samples of nymphal and adult *S. obtrusum* at the Pierce Cedar Creek Institute (PCCI: 42°39′N, 85°17′W), nature preserve in southwest Michigan, during the summers of 2012 and 2013 (Figure 1). In both years, we used D‐nets to till the substrate at wetland sites to collect nymphs. Nymphs were identified to species with a dichotomous key obtained from the Michigan Odonata survey online database (Bright & O'Brien, 2008). Additionally, we sequenced 24 nymphs at the 18S ribosomal RNA gene (Pilgrim & Von Dohlen, 2007; following their methods) as a check on our species identification. We sampled adults using sweep nets when they began to emerge, between 19–27 June 2012 and 24 June to 11 July 2013, and collected the middle right leg from each adult sampled, preventing resampling in the field (Hadrys, Schroth, Schierwater, Streit, & Fincke, 2005). Adults were classified to the species level using a field guide for both years (Nikula, Sones, Stokes, & Stokes, 2002). Nymphs and legs collected from adults were stored in 70% ethanol until DNA extraction.

We sampled nymphs and adults at four aquatic locations across the preserve, along with accompanying nearby prairies (120+ person sampling hours in each habitat type over at least 10 days each year). We collected samples from two lakes, Aurohn Lake (AUR), a man‐made, spring‐fed lake, and Brewster Lake (BRW), a large natural lake. In addition, we collected from Hyla Wetlands (HYL), which includes a small pond, and Tallgrass Swamp (TAL), which is a temporary, seasonal wetland that does not experience wet conditions in every year. These sites represented all potential sites for dragonfly natal waters on the property owned by the institute. TYL, HYL, and BRW were clustered closer to each other in relation to AUR, allowing us to examine movement at different distances within our metapopulation. We expected that the more distant AUR would exhibit greater isolation from the other natal populations. All sites were sampled in both years, but individuals were not found at all sites in both years. We genetically sampled 29 nymphs and 19 adults from AUR in 2012 and 17 nymphs and 22 adults in 2013. From TAL, we sampled 23 nymphs and 16 adults in 2012. Because the Tallgrass swamp had dried up, we found no nymphs in summer 2013, but still sampled 29 adults from the prairie. We sampled 28 nymphs and 22 adults from HYL in 2013, although none were found in the area in 2012. Finally, we genetically sampled 24 adults from BRW in 2012 and 17 in 2013. We found no evidence of nymphs within Brewster Lake. In total, we genotyped 97 nymphs and 149 adult dragonflies from across the PCCI property. Although sample sizes were small, we feel sampling accurately represented the available individuals based on the number of samples collected and sampling efforts (Hale, Burg, & Steeves, 2012).

### 2.2 DNA extraction and AFLP genotyping

We extracted DNA from the abdomen of each nymph and the leg of each adult sampled using DNeasy Tissue Kits (Qiagen Inc., Valencia, CA). We analyzed the samples using amplified fragment‐length polymorphisms (AFLPs) according to the Plant Mapping Protocol (Life Technologies, Corp., Carlsbad, CA), but increased the number of cycles to 30 for the selective amplification polymerase chain reaction (PCR) to increase the number of amplicons. We used the enzymes Mse1 and EcoR1. We used one primer‐pair combination, E‐TAG with M‐CGC (Eurofins MWG Operon, Huntsville, AL) with the selective EcoR1 primer tagged with fluorescent dye (5~HEX), to amplify DNA for PCR. The PCR products were analyzed with an automated DNA sequencer (3130 Genetic Analyzer; Life Technologies, Inc., Foster City, CA), and the bands were scored using GeneMapper 4.0 software (Life Technologies, Inc.). We only accepted peaks that were between 50 and 400 base pairs in size and had a height that was above 70 fluorescent units. For each individual, we scored each band as present (1) or absent (0).

### 2.3 Analysis of population structure

To determine whether nymphs and adults sampled from individual locations in the same year should be analyzed together or separately, we evaluated the genetic similarity between nymphs and adults collected from the same site within the same year using Nei's genetic identity and F_ST_ values, as calculated in AFLP Surv (Vekemans, 2002). Nei's genetic identity ranged from 0.005 to 0.04, while *F*_ST_ values ranged from 0.07 to 0.35 (Supporting Information Table S1). Given the relatively high genetic differentiation by location and sampling year for some comparisons, we treated nymphs and adults from all sampling locations and years as separate entities in all further analyses.

Individuals were grouped according to genetic similarity using the Bayesian analysis program STRUCTURE, which assigns individuals probabilistically to genetic clusters based on allelic frequencies at each locus (Pritchard, Stephens, & Donnelly, 2000). We ran individuals from each of the sampling locations in each year as putative populations to determine clustering across locations and years. We conducted five runs of *K* = 1–24, which allowed for the possibility that each of the sampling locations by year and stage might break down into two separate genetic clusters. We ran the model with 100,000 Markov chain Monte Carlo repetitions and a burn‐in period of 100,000 under the admixture model with independent allele frequencies, as AFLP bands are independent of each other. The optimal value of *K* was evaluated using Ln probabilities and Evanno's Δ*K* (Evanno, Regnaut, & Goudet, 2005; Pritchard, Wen, & Falush, 2009), visualized, and calculated in STRUCTURE HARVESTER (Earl & von Holdt, 2012). Q‐plots of all *K* populations were visualized using CLUMPAK (Kopelman, Mayzel, Jakobsson, Rosenberg, & Mayrose, 2015).

In addition to evaluating genetic clusters in STRUCTURE, we sought to determine how tightly clustered dragonfly nymphs and adults were by location and life stage. We examined differentiation and grouping of individuals using nonmetric multidimensional scaling (NMDS), which allows for the visual representation of individuals grouped, in this case, on genetic parameters. NMDS uses the Sorenson–Bray–Curtis distance measure, which is appropriate for data that does not meet assumptions of multivariate normality, as is the case with presence–absence data. We determined the strength of these groups using multiresponse permutation procedures (MRPP). Both analyses were performed in PC‐ORD (McCune & Mefford, 2011). In addition to *p*‐values, PC‐ORD generates T and A values for all comparisons in MRPP. T is a measure of separation between groups, with more negative values indicating stronger separation. Group homogeneity is described by A and is scaled between 0 and 1.

### 2.4 Genetic differentiation

We calculated the number of unique bands in each cluster determined by STRUCTURE using R version 3.4.0 (R Core Team 2017, Vienna, Austria). We utilized dplyr package 0.7.1, which provides flexible data management strategies for data frames and builds off the package plyr (Wickham, Francois, Lionel, & Müller, 2017). Package plyr provides tools for breaking down data analysis into manageable, manipulatable steps (Wickham, 2011). We randomly sampled individuals from larger groups down to the size of the smallest cluster using the plyr package, with 1,000 repetitions. We compared the number of bands present amongst genetic clusters using an ANOVA, followed by pairwise t tests for each genetic cluster carried out in the data analysis package of Excel 2016. T tests were evaluated with a Bonferroni‐corrected alpha for multiple comparisons. In addition, we calculated F_ST_ and pairwise values of Nei's coefficient of genetic diversity using the program AFLP Surv 1.0 (Vekemans, 2002) between each genetic cluster to better understand genetic differentiation of subpopulations. We ran 100 permutations to test for significance at *ɑ* = 0.05. We used the same methods to evaluate genetic diversity between life stages collected each year and between all sites and stages.

## 3 RESULTS

### 3.1 Genetic clusters

**Figure 2:**
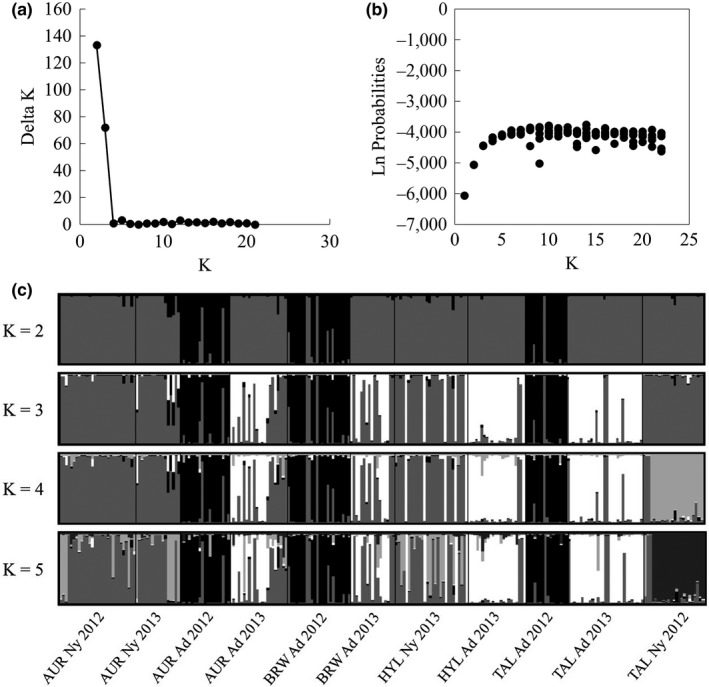
Results of the Bayesian cluster analysis STRUCTURE for each sampling location on PCCI property. (a) Graph of Evanno's Δ*K* for each value of k calculated in STRUCTURE. (b) Graph of Ln Probabilities for each *k* calculated in STRUCTURE. (c) Q‐plot of *Sympetrum obtrusum* genetic clusters assigned by STRUCTURE for *K* = 2–5. Each color represents a unique genetic cluster. In the Q‐plots, each individual is represented by a vertical bar with the colors showing the proportion of the individual genotype derived from respective genetic clusters. Sampling location for both Q‐plots is noted beneath the plot for *K* = 5

We successfully amplified 106 polymorphic bands from 246 dragonflies, and all the sequenced nymphs were returned as *S. obtrusum*. Our results from STRUCTURE yielded conflicting results with regard to the number of genetic clusters in our population. According to Evanno's Δ*K*, the most likely number of genetic clusters on the PCCI property was two (Figure 2a), while the Ln Probabilities method suggested by Pritchard et al. (2000) suggests four separate clusters (Figure 2b). Generally, Evanno's method performs well in populations with high genetic differentiation, but it can be conservative, picking the highest level of population structure (Waples & Gaggiotti, 2006). When considering the Evanno suggested *k* = 2, Cluster 1 includes all AUR nymphs; adults collected from BRW, AUR, and TAL in 2013; all HYL individuals; and the TAL nymphs (Figure 2c). Cluster 2 includes adults from AUR, BRW, and TAL in 2012 (Figure 2c). Under the solution *k* = 4, as suggested by Pritchard's method, Cluster 2 remains intact, but Cluster 1 breaks into three additional clusters (Figure 2c). The composition of these clusters aligns closely with clusters detected by a hierarchical analysis, described below.

**Figure 3:**
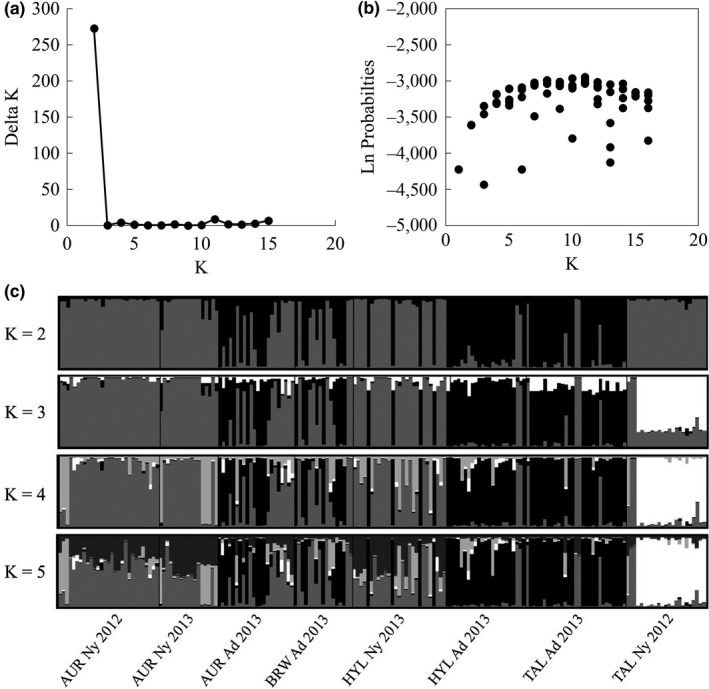
Results of the hierarchical Bayesian cluster analysis STRUCTURE for each sampling location on PCCI property found in the 1st suggested cluster of the original STRUCTURE analysis. (a) Graph of Evanno's Δ*K* for each value of k calculated in STRUCTURE. (b) Graph of Ln Probabilities for each *k* calculated in STRUCTURE. (c) Q‐plot of *Sympetrum obtrusum* genetic clusters assigned by STRUCTURE for *K* = 2–5. Each color represents a unique genetic cluster. In the Q‐plots, each individual is represented by a vertical bar with the colors showing the proportion of the individual genotype derived from respective genetic clusters. Sampling locations are noted beneath the plot for *K* = 5

Because the two methods for evaluating STRUCTURE results yielded different numbers of genetic clusters, we performed a hierarchical analysis to detect further division in the two clusters suggested by Evanno's Δ*K*. The more conservative Evanno's method may underestimate population structure when there is a hierarchical element (Waples & Gaggiotti, 2006). As such, we sought to detect the largest number of clusters with biological significance and based our decisions for the final arrangement of clusters on a combination of Pritchard's method, Evanno's method, and evaluation of the potential biological explanations for each cluster. Based on Pritchard's suggested method (Pritchard et al., 2000), we left the cluster containing the AUR, BRW, and TAL adults from 2012 as a single cluster. The choice of a single cluster was supported when this group of samples was run independently from the other sample locations. We divided the other adults and nymphs into *k* = 3 additional populations based on the results from Evanno's Δ*K* and Pritchard's method for the hierarchal analysis of the relevant sampling locations (Figure 3a and b). In this arrangement, genetic Cluster 1 includes the adults from AUR, BRW, and TAL collected in 2012. Cluster 2 includes AUR nymphs, most HYL nymphs, and some adults collected in AUR and BRW in 2013 (Figure 3c). Cluster 3 includes the remaining AUR and BRW adults from 2013, as well as the adults from HYL and TAL collected in 2013 (Figure 3c). The final genetic cluster, Cluster 4, includes only the TAL nymphs collected in 2012 (Figure 3c).

**Figure 4:**
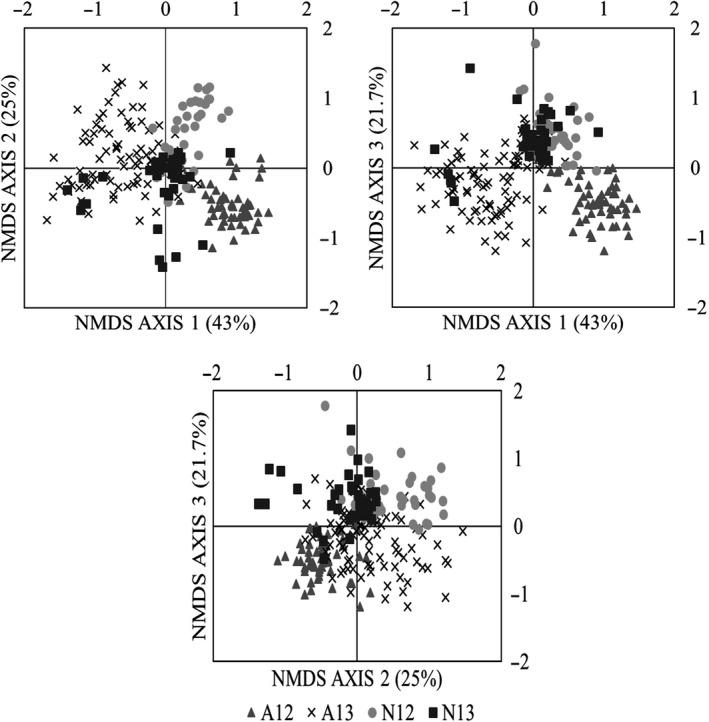
Plots of NMDS scores from AFLP bands for individual dragonflies sorted by life stage and year captured. Monte Carlo tests of randomization suggested that a three‐dimensional solution best explained the data. Axis 1 explains 43% of the variation in the data, Axis 2 explains 25% of the variation in the data, and Axis 3 explains 21.7% of the variation in the data. Each plot represents a pairwise comparison of scores from two of the three axes, with all combinations shown. All plots follow the same legend and are coded as follows: A12: 2012 Adults; A13: 2013 Adults; N12: 2012 Nymphs; and N13: 2013 Nymphs

We completed nonmetric multidimensional scaling (NMDS) analysis of dragonflies at PCCI based on location and year, as well as life stage and year (Figure 4). Monte Carlo tests of randomization suggested that a three‐dimensional solution would provide the best explanatory power for our data. The final stress for a three‐dimensional solution was 13.4, while the stress for a two‐dimensional solution was 18.7, and the stress for a one‐dimensional solution was 32.9. Under a three‐dimensional solution, 106 AFLP bands were condensed to three axes, explaining 43%, 25%, and 21.7% of the variation in the data, totaling 89.7%.

Overall, sampling locations from each year and life stage were significantly different from each other (*T* = −65.3, delta = 0.042, A = 0.311, *p* < 0.001). Amongst the 55 pairwise comparisons, almost all sampling locations differed significantly from each other after Bonferroni correction (See Supporting Information Table S1). Values of T ranged from −0.159 to −28.8, with all values for significant comparisons less than −6.49. Values of A ranged from 0.002 to 0.354, with all values for significant comparisons greater than 0.042. There were no significant differences in pairwise comparisons of the adults collected in 2012 from AUR, BRW, and TAL from each other. Additionally, the HYL nymphs were not significantly different from the AUR nymphs or adults collected in 2013 nor were the AUR adults from 2013 different from the BRW adults collected in 2013. All other pairwise comparisons were significantly different.

We also coded the NMDS scores for individual dragonflies based on life stage, grouping them into 2012 nymphs, 2012 adults, 2013 nymphs, and 2013 adults (Figure 4). In MRPP analysis, life stage groups were significantly different from each other (*T* = −92.9, delta = 0.046, A = 0.239, *p* < 0.0001). In pairwise comparisons, all life stage groups were significantly different from each other. Pairwise T values ranged from −18.6, for a comparison of nymphs in both years, to −78.3, for a comparison of adults in both years. Pairwise A values ranged from 0.057, again for the comparison of nymphs in both years, to 0.235 for a comparison of adults in both years. Pairwise comparisons involving adults sampled in 2012 had consistently high A values and more negative T values than comparisons not involving those adults (See Supporting Information Table S2). The 2012 adults showed the strongest differentiation and separation from all other groups, while the nymphs of both years showed the least differentiation from each other.

### 3.2 Unique genetic variation and genetic differentiation

We found a significant difference in the number of unique bands sampled across genetic clusters, when corrected for sample size (*F* = 141.3, *p* < 0.0001). All pairwise comparisons were significantly different (*p* < 0.001) at a Bonferroni‐corrected alpha of 0.008. The highest number of unique bands (9.5) was found in Cluster 2, followed by Clusters 4 and 3, with 9.2 and 8.7, respectively. The lowest number of unique bands (7.5) was found in Cluster 1, which contained 2012 adults.

When all sites, separated by collection year and life stage, were compared individually, *F*_ST_ was 0.2219. Pairwise *F*_ST_ values ranged from 0.001 to 0.333 and Nei's coefficient of genetic identity ranged from 0.001 to 0.049. All comparisons were significantly different with the exception of the pairs identified as similar by the MRPP analysis above. Across the four genetic clusters identified by STRUCTURE, *F*_ST_ was 0.2557. Pairwise *F*_ST_ values ranged from 0.15 to 0.326 (*p* < 0.001), while pairwise values for Nei's Coefficient of Genetic Identity ranged from 0.01 to 0.41 (Table 1). For the overall comparison of adults and nymphs collected in 2012 and 2013, *F*_ST_ was 0.2198. Pairwise *F*_ST_ values ranged from 0.058 to 0.305 (*p* < 0.001), while pairwise Nei's values ranged from 0.003 to 0.34 (Table 2).

**Table 1:** Pairwise *F*_ST_ values (above the diagonal) and pairwise Nei's Coefficient of Genetic Identity (below the diagonal) for four genetic clusters of *Sympetrum obtrusum*, as identified by STRUCTURE

	Cluster 1	Cluster 2	Cluster 3	Cluster 4
Cluster 1		0.306	0.297	0.326
Cluster 2	0.030		0.168	0.150
Cluster 3	0.041	0.015		0.225
Cluster 4	0.039	0.010	0.025	

*Note*

Descriptions of clusters are provided in the text. All pairwise comparisons of *F*_ST_ were significant (*p* < 0.001).

**Table 2:** Pairwise *F*_ST_ values (above the diagonal) and pairwise Nei's Coefficient of Genetic Identity (below the diagonal) for the two life stages sampled in each year

	Adults 2012	Adults 2013	Nymphs 2012	Nymphs 2013
2012 Adults		0.279	0.305	0.291
2013 Adults	0.034		0.126	0.117
2012 Nymphs	0.032	0.011		0.058
2013 Nymphs	0.030	0.009	0.003	

*Note*

All pairwise comparisons of *F*_ST_ were significant (*p* < 0.001).

## 4 DISCUSSION

The population of *S. obtrusum* at PCCI appears to exist as a strongly differentiated metapopulation structure. STRUCTURE detected four genetic clusters across the area. These genetic clusters were grouped not only by location, but also by collection year, with all adults collected in 2012 grouping distinctly from all other groups. The remaining groups were mixes of adults and nymphs collected in 2012 and 2013, with the exception of the final group, which contained only the nymphs from Tallgrass Swamp collected in 2012. In terms of F_ST_, the clusters were moderately to strongly differentiated, suggesting low exchange of individuals between these genetic clusters. Pairwise comparisons of all individual sampling locations loosely confirm these findings. Once again, the 2012 adult populations were not significantly different from each other but were significantly different from all other locations.

The results of the NMDS and MRPP analyses followed the same general patterns at the structure analysis. Pairwise comparisons showed the same statistical patterns as pairwise *F*_ST_ tests. When grouped by life stage and year collected, the 2012 adults were most strongly differentiated from all other groups. The nymphs collected in 2012 and 2013 were the least differentiated from each other and from all other groups. This suggests that nymphs in this population experience variable timing of emergence, as might be expected given that semivoltinism can vary due to environmental conditions (Corbet, 1999). Dragonflies, including *Sympetrum* species, may overwinter for multiple years prior to transitioning from nymph to adult dragonflies, creating overlapping generations within the same emergence (Kormondy & Gower, 1965; Pintor & Soluk, 2006). The results from the STRUCTURE analysis support this finding by grouping the 2012 and 2013 nymphs into the same genetic cluster. The genetic cluster containing most of the 2012 and 2013 nymphs also included some 2013 adults, but not all, suggesting that some of the nymphs from 2012 emerged at PCCI in 2013, while others remained in the water for at least an additional year. However, the remaining adults sampled in 2013 represented genetic information not generally found in the nymphs, or in the 2012 adults, suggesting immigration of adults that emerged from other water sources.

Variable semivoltinism could explain some of the patterns observed in the metapopulation of *S. obtrusum*. However, other mechanisms, including adult dispersal and breeding behavior, are likely at play in the population. Our results support findings that adult *S. obtrusum* move away from their natal body of water to forage after emergence. The movement of adult dragonflies to a new area for breeding and foraging supports previous mark–recapture studies which showed high percentages of nonlocal dragonflies at closely aggregated ponds (Michiels & Dhondt, 1991); however, the movement distance varies largely between dragonfly species (Conrad et al., 1999). Unfortunately, we cannot definitively determine, based on our data alone, whether they are also mating away from their natal ponds. However, the strong genetic differentiation of the 2012 adults from any adults or nymphs sampled in 2013 suggests that those adults likely bred elsewhere. The ponds and swamps at our site are between 1 and 2 km apart, but there are several other privately and publicly owned bodies of water in the area, including some within 1 km of the lakes we sampled, that could serve as breeding locations for adult dragonflies sampled within the study area. Previous studies have suggested that some dragonfly species show very little dispersal out of their natal ranges, especially beyond 1 km (Angelibert & Giani, 2003; Keller et al., 2010); however, studies of *Sympetrum* species have shown that up to 47% of adults move away from their natal pond when other ponds were less than 1 km apart (Conrad et al., 1999). This behavior may vary heavily by species, abiotic factors, and age or sex of individuals in the population though (Angelibert & Giani, 2003; Dolný et al., 2013). In addition, dragonflies may utilize a terrestrial area 1,000 times the size of their aquatic natal area (Dolný et al., 2014).

The differentiation of sampling locations in pairwise comparisons suggests that, although *S. obtrusum* have the capacity to fly great distances and utilize large home ranges, their ability to reach different locations on the landscape may be hindered by factors other than distance. For instance, the assemblage of dragonfly species at ponds is influenced by agricultural land management, as mowing may cause direct mortality and reduce available roosting habitat (Raebel et al., 2012). In addition to land use practices, roads also may alter dragonfly movement. For some species, this effect will be negative as a result of direct mortality, while other species utilize roadways as corridors between noncontiguous habitats (Soluk, Zercher, & Worthington, 2011). PCCI is a mosaic of farmland and forest habitat intersected by local roads. While many of the adults fall into similar genetic clusters, there is still a great deal of genetic differentiation between sampling locations, suggesting limitations to movement across the landscape.

Finally, the dragonfly metapopulation structure at PCCI may be influenced by *Sympetrum* breeding biology. Dragonflies are known to mate with multiple individuals. Although male dragonflies are often territorial and practice mate guarding to prevent females from mating with other males, some males do fail to completely protect their females from intruding males. Likewise, some males will mate with additional females (Harvey & Hubbard, 1987). In addition, females may be forced by male harassment to cease oviposition and change locations before beginning again (McMillan, 2000). These two forces may combine to increase intermixing between locations, explaining some of the lack of differentiation between nymphs on the PCCI property.

Understanding the patterns of population structure and dragonfly movement within a metapopulation can have important implications for dragonfly conservation. Anthropogenic land use can drastically alter landscapes, removing critical habitat for both nymphs and adult dragonflies. The loss of individual foraging grounds or natal waters could represent a disruption of metapopulation structure and the loss of unique genetic material in the overall population. In our study, the potential for the loss of unique genetic information was evidenced by the loss of the Tallgrass Swamp population. Nymphs were sampled in the swamp in 2012, but the swamp dried up prior to sampling in 2013. The nymphs sampled from this area in 2012 represented a unique genetic cluster and contained the second highest number of unique AFLP bands in the population. While adults were sampled in the nearby prairie in both 2012 and 2013, they were not part of the same genetic cluster as the nymphs, again suggesting movement away from the natal area, at least for foraging. Although we were unable to sample any eggs or larvae from Tallgrass swamp in 2013, it is possible that the genetic material from these nymphs survives in adults elsewhere. As part of the flexible semivoltine strategies exhibited by dragonfly populations, *Sympetrum* species have an evolutionary history of using temporary and annual pools for egg laying (Corbet et al., 2006), with eggs able to survive at least 8 months in dried pools (Wiggins, Mackay, & Smith, 1980). This strategy may provide insurance for maintaining metapopulation connectivity and genetic diversity in spite of the fragile nature of some of the pools in which *Sympetrum* lay their eggs. However, for long‐term metapopulation survival, the maintenance of landscape connectivity is crucial for maintaining genetic diversity in dragonfly metapopulations, especially in the face of climatic change and anthropogenic land use changes.

Habitat degradation, including the loss of connected habitat, is a leading force in recent declines in local biodiversity (Pimm & Raven, 2000). Changes in human land use patterns have altered habitat at local and global scales, often leading to declines in biodiversity. In particular, in freshwater habitats, water quality and availability may be degraded by agriculture or urbanization (Foley et al., 2005). These patterns of land use degradation put dragonfly species at risk, especially in light of the patterns of population connectivity observed in this study. The loss of habitat and connectivity may potentially disrupt the breeding cycle and movement patterns of dragonflies, leading to losses of genetic diversity and potentially to the loss of populations. Given the increases in land alterations by humans, managers should be vigilant in maintaining habitat for vulnerable species, including dragonflies.

## CONFLICT OF INTEREST

None declared.

## AUTHOR CONTRIBUTIONS

Payton Phillips analyzed data and wrote the manuscript. Bradley J. Swanson designed the research, supervised the fieldwork, provided guidance on data analysis, and edited the manuscript.
