# Supplementary material for: A genetic analysis of dragonfly population structure
Source: Ecol Evol. 2018 Jun 25;8(14):7206–15. doi: 10.1002/ece3.4255 (PMC6065342; doi:10.1002/ece3.4255)
Supplement: Supplementary file 1 [file ECE3-8-7206-s001.pdf]

Table S1: Pairwise comparisons of sampling locations by year under MRPP, F_{ST}, and Nei's genetic distance. We use a Bonferroni correction for multiple tests (α = 0.0009). T and A are measures associated with MRPP. T is a measure of separation between groups, with more negative values indicating stronger separation. A describes within group homogeneity on a scale between 0 and 1. For F_{ST}, p was calculated with 500 permutations and the alpha corrected for multiple tests (α = 0.0009). All p-values are significant except those indicated by [A].

Pop1	Stage	Pop2	Stage	T	A	p	Nei's	F_{ST}	p
AUR	12N	AUR	13N	-10.3	0.076	8.60E-07	0.003	0.060	<0.001
		AUR	12A	-28.2	0.347	<0.001	0.037	0.345	<0.001
		AUR	13A	-17.7	0.114	<0.001	0.006	0.083	<0.001
		BRW	12A	-27.4	0.303	<0.001	0.028	0.296	<0.001
		BRW	13A	-17.6	0.129	<0.001	0.006	0.100	<0.001
		HYL	13N	-11.4	0.057	1.00E-08	0.002	0.045	<0.001
		HYL	13A	-27.5	0.236	<0.001	0.022	0.210	<0.001
		TAL	12N	-24.1	0.19	<0.001	0.011	0.166	<0.001
		TAL	12A	-26.3	0.354	<0.001	0.039	0.358	<0.001
		TAL	13A	-28.8	0.248	<0.001	0.022	0.225	<0.001
AUR	12A	AUR	13N	-18.5	0.286	1.00E-08	0.035	0.317	<0.001
		AUR	13A	-22.3	0.268	<0.001	0.036	0.291	<0.001
		BRW	12A	-5.38	0.049	0.001[A]	0.005	0.055	0.018[A]
		BRW	13A	-19.8	0.296	<0.001	0.038	0.311	<0.001
		HYL	13N	-24.8	0.249	<0.001	0.034	0.302	<0.001
		HYL	13N	-24	0.306	<0.001	0.048	0.315	<0.001
		TAL	12N	-24.1	0.33	<0.001	0.043	0.336	<0.001
		TAL	12A	-0.159	0.002	0.369[A]	0	0.001	0.447[A]
		TAL	13A	-26.8	0.319	<0.001	0.048	0.327	<0.001
AUR	13N	AUR	13A	-10.9	0.096	3.50E-07	0.005	0.074	<0.001
		BRW	12A	-16.9	0.224	6.00E-08	0.024	0.254	<0.001
		BRW	13A	-8.87	0.097	5.37E-06	0.005	0.073	<0.001
		HYL	13N	-3.2	0.024	0.011[A]	0.001	0.012	0.02[A]
		HYL	13A	-18	0.193	<0.001	0.021	0.198	<0.001
		TAL	12N	-16.3	0.177	1.00E-08	0.012	0.16	<0.001
		TAL	12A	-16.7	0.292	6.00E-08	0.035	0.321	<0.001
		TAL	13A	-19.3	0.198	<0.001	0.022	0.212	<0.001
AUR	13A	BRW	12A	-21.1	0.219	<0.001	0.025	0.231	<0.001
		BRW	13A	-2.53	0.02	0.022[A]	0.001	0.011	0.074[A]
		HYL	13N	-6.49	0.042	<0.001	0.003	0.037	<0.001
		HYL	13A	-7.3	0.051	2.86E-05	0.007	0.068	<0.001

		TAL	12N	-19.6	0.163	<0.001	0.013	0.144	<0.001
		TAL	12A	-20.5	0.271	<0.001	0.037	0.299	<0.001
		TAL	13A	-10.6	0.073	1.93E-06	0.009	0.088	<0.001
BRW	12A	BRW	13A	-19	0.236	1.00E-08	0.027	0.251	<0.001
		HYL	13N	-23.4	0.212	<0.001	0.024	0.246	<0.001
		HYL	13A	-24.9	0.268	<0.001	0.037	0.269	<0.001
		TAL	12N	-25.4	0.303	<0.001	0.035	0.302	<0.001
		TAL	12A	-2.14	0.022	0.04[A]	0.001	0.012	0.210[A]
		TAL	13A	-27.8	0.291	<0.001	0.037	0.278	<0.001
BRW	13A	HYL	13N	-6.89	0.049	<0.001	0.003	0.044	<0.001
		HYL	13A	-10.8	0.091	4.00E-07	0.01	0.1	<0.001
		TAL	12N	-18.2	0.19	<0.001	0.015	0.171	<0.001
		TAL	12A	-18.2	0.307	3.00E-08	0.039	0.321	<0.001
		TAL	13A	-11.2	0.095	1.53E-06	0.011	0.111	<0.001
HYL	13N	HYL	13A	-19.1	0.142	<0.001	0.017	0.164	<0.001
		TAL	12N	-20.2	0.143	<0.001	0.011	0.139	<0.001
		TAL	12A	-23	0.251	<0.001	0.035	0.312	<0.001
		TAL	13A	-16.3	0.132	1.20E-07	0.016	0.16	<0.001
HYL	13A	TAL	12N	-24.2	0.218	<0.001	0.025	0.209	<0.001
		TAL	12A	-22.4	0.311	<0.001	0.049	0.325	<0.001
		TAL	13A	-16	0.104	<0.001	0.012	0.101	<0.001
TAL	12N	TAL	12A	-22.4	0.336	<0.001	0.044	0.348	<0.001
		TAL	13A	-27.5	0.273	<0.001	0.032	0.265	<0.001
TAL	12A	TAL	13A	-24.8	0.32	<0.001	0.049	0.333	<0.001

Table S2: MRPP pairwise comparisons of dragonflies by year. Significant p-values after Bonferroni correction for multiple tests ($\alpha = 0.0083$) are shown with an asterisk. T is a measure of separation between groups, with more negative values indicating stronger separation. A describes within group homogeneity on a scale between 0 and 1.

Compared	T	A	p
2012 Adults vs. 2013 Adults	-78.3	0.235	<0.001*
2012 Adults vs. 2012 Nymphs	-58.2	0.270	<0.001*
2012 Adults vs. 2013 Nymphs	-50.0	0.233	<0.001*
2013 Adults vs. 2012 Nymphs	-48.1	0.116	<0.001*
2013 Adults vs. 2013 Nymphs	-29.3	0.074	<0.001*
2012 Nymphs vs. 2013 Nymphs	-18.6	0.057	<0.001*
